# Primary healthcare reform for chronic conditions in countries with high or very high human development index: A systematic review

**DOI:** 10.1177/17423953211059143

**Published:** 2021-11-29

**Authors:** Mohammed Alyousef, Corina Naughton, Colin Bradley, Eileen Savage

**Affiliations:** 1 School of Nursing and Midwifery, 8795 University College Cork, Cork, Ireland; 2 Department of Health Services Administration and Hospitals, King Abdulaziz University, Jeddah, Saudi Arabia; 3 The Department of General Practice, 8795 University College Cork, Cork, Ireland

**Keywords:** Primary healthcare, integrated care, health services, healthcare reform, chronic disease management

## Abstract

**Objective:**

To address the growing burden of chronic disease globally, many countries have developed a national policy for primary healthcare reform. In some countries with high and very high human development index, evaluations of the implementation of these reforms have been published. To date, there has been no systematic review of these evaluations. The objectives of this review are to identify: (a) the vision for primary healthcare; (b) the features of primary healthcare reforms; and (c) evaluation findings of primary healthcare reforms.

**Methods:**

A systematic literature review was conducted guided by the PRISMA statement. We searched for academic articles and grey literature from 1 March 2008 to 1 September 2020. Screening and data extraction were conducted by two authors. Descriptive analysis and narrative synthesis were applied.

**Results:**

A vision for integrated primary healthcare shifting chronic disease management from specialist hospital services to primary care was found to require new organization and funding models such as collaborative primary healthcare networks and commissioning along with shared governance across health sectors. The need for general practitioner leadership and engagement to support primary healthcare reform was identified. Although there was evidence of barriers in progressing primary healthcare reform, evaluation results showed some positive outcomes, most notably shifts in services towards increased primary care access and utilization.

**Discussion:**

A challenge in undertaking the review was the heterogeneity of articles with little consistency in how primary healthcare reform was evaluated and reported on across countries. Evaluation of national health reforms involves complex system-wide projects and is an area that needs further exploration and discussion to determine the most appropriate methodologies for collecting and analysing large-scale data with consideration for service and health outcomes.

## Introduction

Globally, chronic disease and multimorbidity represent major challenges, increasing the health demand in many countries.^1^ The World Health Organization (WHO)^2^ reported that 15 million people aged 30–69 years old die each year from non-communicable diseases, and this number is expected to rise to an estimated 52 million by 2030. In responding to the burden of chronic disease, the WHO *Global Status Report on Non-Communicable Diseases 2010*^3^ highlighted the importance of strengthening the health system with more emphasis on primary healthcare (PHC) as the first point of care and partnership between health services providers as key to success. Effective chronic disease management (CDM) has been linked with strong PHC characterized by good governance structures, accessibility, care coordination, continuity of care and comprehensive service delivery.^1^ Hone et al.^4^ indicated that countries with stronger PHC have been able to reduce mortality rates, part of which can be attributed to effective management of non-communicable diseases.

In an effort to tackle major deficits in health systems such as fragmentation, inequalities and inefficiencies, many countries have been undergoing reform of PHC including integration between primary and secondary healthcare services.^5–7^ CDM is a priority in these reforms shifting from predominately secondary specialist care to PHC as the principal setting for accessing and receiving healthcare.^5–7^ The Health Council of Canada reported that effective PHC reform will achieve enhanced continuity of care through integrated healthcare; a shift in health service provision from individuals to populations; more efficient use of qualified professional health workers leading to effective cooperation between health providers; a focus on disease prevention, and health promotion and more appropriate use of healthcare resources.^8^ It has been suggested that chronic diseases and long-term health conditions are more effectively managed in countries with high or very high human development index (HDI) due to more advanced healthcare systems compared to those with low or medium HDI.^9,10^ Therefore, countries with high/very high HDIs may offer insights into the impact of PHC reforms on CDM. The HDI, as reported annually by the United Nations Development Programme, is a measure of a country's human development in terms of a long and healthy life expectancy, mean years of education, and gross national income per capita contributing to standards of living.^11^

Conceptually, PHC is broader than primary care (PC),^12^ yet, these concepts are used interchangeably leading to confusion.^12,13^ PC is a component of PHC referring to health services delivered to individuals closest to their communities through family doctor type services known as general practices (GPs) or family practices.^12–14^ More broadly, PHC, which focuses on population health, is viewed as a complex subsystem within a country's national health system. It incorporates a wide range of public health, primary and community services.^13^

In strengthening PHC reform for CDM, the WHO^3^ highlighted the need to evaluate progress at the national level. An evaluation of these reforms would help to determine the vision for PHC reform for the prevention and control of non-communicable chronic diseases, as well as the features of these reforms in terms of the services developed and implemented for CDM.^3^ To date, a number of countries have reported on evaluations of PHC reform national policy concerning CDM. However, there has been no systematic review of these national evaluations. A review of this evidence would help to identify the vision and features of PHC reform targeting CDM, and a synthesis of evaluation findings would help to determine what works best and why. This systematic review aims to address this gap in knowledge. With a focus on countries with high or very high HDI, the objectives of this review are to identify: (a) the vision for PHC reform relating to CDM; (b) the features of PHC reforms in terms of the services developed and implemented for CDM; and (c) evaluation findings of PHC reforms for CDM.

## Methods

This systematic review was guided by the PRISMA statement.^15^ Consistent with the review objectives, the search process and selection criteria were guided by the following research questions: What is the vision for PHC reform relating to chronic diseases? What are the features of PHC reform relating to services developed and implemented for CDM? What are the outcomes of evaluations of national PHC reform targeting CDM?

### Search strategy

We searched the academic databases CINAHL, MEDLINE and Embase to identify articles focusing on PHC reform targeting chronic diseases. We applied the terms ‘primary healthcare’, ‘primary care’ and ‘community’ since these terms are often used interchangeably in the literature. Other terms included ‘health services/system reform’ and terms relating to chronic conditions such as ‘chronic disease’/’chronic condition’/ ‘non-communicable disease’. In addition, we applied the term ‘reform’. Boolean operators (OR/AND) as well as truncation where relevant were applied. The relevant subject headings, MeSH terms and index terms were applied, respectively, to CINAHL, MEDLINE and Embase databases. The search was limited by date from 1 March 2010 to 1 September 2020 reflecting articles published since the WHO emphasized strengthening the health system through PHC in responding to the burden of chronic disease.^3^ The search was also limited to articles published in the English language. A search for grey literature was conducted guided by Godwin et al.^16^ Initially, three databases/websites were searched, namely OpenGrey, Grey Literature Report (up to 2017 when discontinued) and the WHO. The second search was then conducted in targeted websites of countries shown to yield a greater volume of articles retrieved from these sites and the academic databases. Websites searched were the National Health Services, Department of Health and The Kings Fund from the UK, Health Canada, Australian Government Department of Health, Ministry of Health New Zealand and the National Institute of Health, USA.

### Selection criteria

Guided by our research questions stated above, we included articles on the evaluation of PHC reform in healthcare systems as part of government strategy in countries with high or very high HDI scores (i.e. index of 7–10) reported for the year(s) concerning the period of evaluation addressed and not the year of publication (sourced through http://hdrundp.org/en/countries). Articles focusing on physical chronic diseases were included since these contribute to a significant burden on health systems due to high morbidity and mortality rates.^1^ Articles of any study design were included, for example, cross-sectional studies, cohort studies, surveys, case studies or qualitative evaluation studies. Additionally, the review included any type of report such as case reports, commissioned reports and narrative reviews. This review excluded articles on countries with low or medium HDI scores. Articles addressing national reform strategy and associated PHC were excluded if the focus was on an individual chronic disease rather than multiple chronic diseases since our aim was to review PHC reform regarding chronic diseases in general. Given the focus of this review on physical chronic conditions, articles on mental illness as a chronic condition were excluded. Furthermore, we excluded articles relating to clinical care provided by family doctors/GPs if not related to the broader government context of health system reform in relation to population health for CDM. Finally, any article reporting on PHC reform for years within which a country did not meet the HDI inclusion criteria was excluded, regardless of the article being published within the time limits set for this review. For example, one article retrieved was published in 2015 from China^17^ but the time period of evaluating PHC reform was 2011–2013 during which time this country was classified as medium human development.^18^

### Screening process

**Figure 1.**
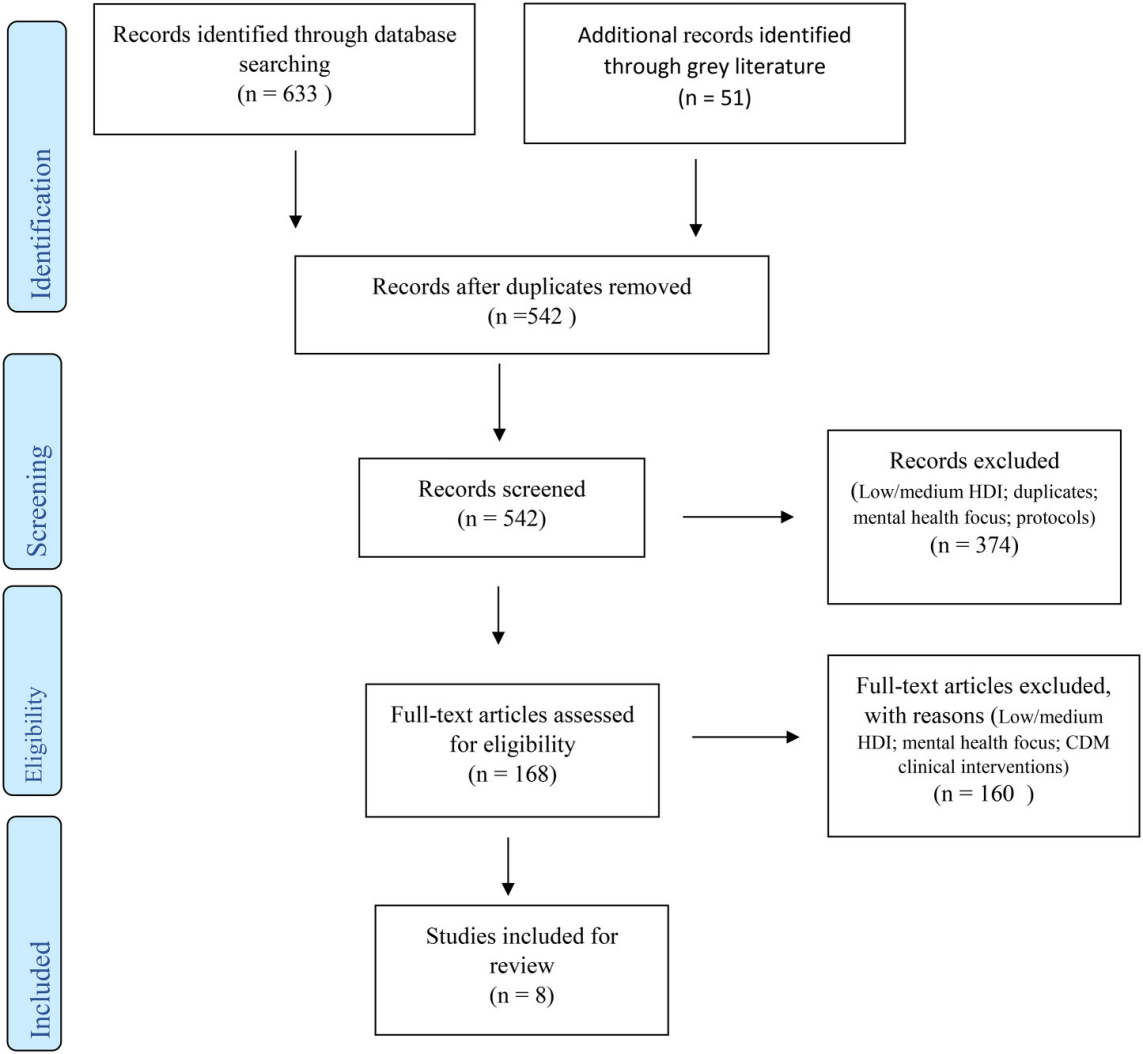
PRISMA flow chart.

All records from the search strategy were first exported to Endnote X7 and then to Covidence for screening once duplicates (n = 142) were removed. Two reviewers (MA, ES) screened the titles and abstracts of each article (n = 542) to determine if full-text review was needed. Full-text articles (n = 168) were then read to establish eligibility for inclusion. Disagreements were resolved by discussion with the other reviewers (CN, CB). The output from the search strategy and screening process is shown in Figure 1 illustrating that eight articles were included in this review. Of these, five were retrieved from academic databases,^19–23^ and three from grey literature sources.^24–26^

### Data extraction and synthesis

Data from each article were extracted into a table including author name(s), publication date, country, design, the vision for PHC reform, features of PHC reform, sources of evaluation data and the findings on outcomes (Table 1). The data were extracted by MA and cross-checked by ES including discussion between both authors to reach a consensus where differences arose. Following data extraction, a narrative synthesis of the findings was conducted structured around the objectives of the review. Although guided by the PRISMA statement,^15^ a meta-analysis was not feasible due to the heterogeneity of studies included.

**Table 1.** Data extraction table.

Country, publication and study design	Vision of reform	Features of PHC reform	Evaluation data sources	Evaluation findings
Estonia, High HDI Atun et al. 2016^19^ Observational study	A health system that improves population health over 5 years by shifting CDM from hospitals to primary care that is financially sustainable.	Family medical-centred PHC: funding reallocation prioritizing outpatient versus in-patient and PHC services; funding based on capitation and pay-for-performance; nationwide integrated e-health system; expansion of family nursing services in family medical centres; PHC development plan for prevention and CDM in PHC, e.g. clinical guidelines. PHC is viewed as critical. PHC is noted as critical for achieving Universal Health Coverage in Estonia.	35.6 M PHC records, and outpatient and inpatient records CD data (n = 552,822 PTs)	Increase in nurses by 26.7% in family medicine practices. Nurse’s consultation increased fourfold. Total PT contacts increased for PHC (79%–81%), decreased for outpatients (20%–17.5%), in-patients (2.2%–1.4%) care. Increase shift in CDM consultations to PHC for diabetes (58.7%–69.6%), hypertension (88%–90.2%), and ischaemic heart disease (67.2%–69.5%) but not for COPD and heart failure.
Australia, Very high HDI Nicholson et al. 2012^20^ Narrative review	A PHC system within a national health strategy that has general practice at its core striving for accessibility, equitability, and regional integration within and between PHC and other sectors for improved quality, safety, performance and accountability.	Expansion from 2011 of meso-level PHCOs (Divisions of general practice (GP)), i.e. intermediate structures between local GPs and government. PHCOs evolved to larger Medicare locals rolling out 2010 national PHC strategy: commissioning/reallocation of funding; integrated governance between sectors; integrated e-health system (dedicated funding); expansion of PHC teams especially practice nurses; after hours; CDM programmes with funding incentives and quality improvement care collaborative programmes.	Published data on PHC activities	Fragmentation of services due to the absence of integrated governance. 96% of GP practices computerized supporting EHR system. 60% of GP practices employ practice nurses. Visits to GP practices with practice nurses doubled from 4.2% to 9%. Increase in GP practices engaged in care collaborative programmes for diabetes and cardiovascular disease management.
Australia, Very high HDI Reeve et al. 2015^21^ Cross-sectional study	To improve population healthcare and CDM in a remote region for the aboriginal community through more equitable and accessible services.	Comprehensive primary healthcare services partnership model between PHC, hospital and cultural health services with single governance structure for funding; PHC and CDM funding investment; CDM programmes aligned to government strategy Interdisciplinary teams with occasional specialist support in the community. Integrated information system.	Data on all residents (approx. 2664 in 2006; and 2942 in 2012) from multiple sources; data from Department of Health databases, quality-of-care indicators (e.g. glycated haemoglobin level, blood pressure and receiving anti-hypertensive medication).	Growth in PHC funding from 23% to 34%; 60% increase in electronic health records. Team care arrangements increased (0%–78%). Trends for service use increased for PHC and decreased for emergency care and hospital admissions. Increase in health assessments (17%–61%) resulted in 73% identified with diabetes placed on care plan. No significant improvements in glycaemic control for diabetes care. Decline in mortality rates (95% CI: 5.71−13.06 to 0.83−4.60).
Australia, Very high HDI Horvath, 2014^24^ Commissioned review	To shift the healthcare from a siloed, episodic system to a patient-centred intergrated system that is seamless, accessible and cost-effective.	Medicare locals (MLs) established as ‘not for profit’ companies from 2011 as a transition from divisions of GP: government funding allocated to MLs for coordinated PHC in local communities, and broader workforce; some MLs have commissioning role; ML engagement with PHC, community and hospital sectors.	Ernst & Young Analysis and opinion Deloitte & Touche Financial audit Stakeholder submissions (n = 270) Interviews with stakeholders and opinion leaders.	Most MLs lack clarity on purpose and direction. Lack of shared governance leavs GPs disempowered. Deficits in financial performance, e.g. planned-actual budget mismatch, service funding duplications. GPs dissatisfied with after hours scheme, e.g. funding inconsistencies across MLs, contracting complexity, and reporting burden. Continued disjointed and fragmented services with a disconnect between MLs and LHNs, and with private health sector. Episodic approach to CDs services inadequate in addressing complexity and multimorbidity requiring integrated coordinated services. Performance monitoring was input and process data rather than population outcome data that could be achieved with roll out of e-health system. A shift from MLs to new PHC organizational structure needed, referred to as primary health care organizations.
Québec, Very high HDI Pineault et al. 2016^22^ Before-after ‘natural’ experiment	To improve continuity of care, enhance accessibility to services, and to improve the integration of CDM	From the early 2000s, family medicine groups (FMGs) of 6–10 GPs and complementary network clinics (NCs); funding and contractual agreement for service provision; NC with extended practice hours, on-call; walk-in clinics; medical specialists; diagnostic and imaging tests.	Population surveys of PTs with various CDs with data covering 2 years (n = 6198 for 2003–2005) and n = 6753 for 2008–2010 divided into experimental group (EG), i.e. FMGs, NCs, FMG-NCs and control group (CG, i.e. other practices not involved in reform Organization telephone surveys of PHC practices (n = 659 in 2005 and 606 in 2010).	Increased PHC use for both groups (79%–86.5%; *p* = 0.001) Perceived care experience: (a) Accessibility declined in both groups but higher for EG (*p* = 0.004). (b) Continuity of care higher and increased more for CG versus EG (*p* < 0.001). (c) Care outcomes higher and increased for CG versus EG (*p* = <0.001). (d) No significant differences on unmet needs in both groups. (e) Decreased service use for both groups; greater for EG with significant DD value (*p *= 0.049), equating to a 12.7% decrease. (f) No significant differences between groups for ER and hospital admissions. Noted that experiences of care and service use related to physican care and excluded other professionals.
Quebec, Canada, Very high HDI Breton et al. 2015^23^ Descriptive with service case examples	Population-based approach to PHC services for CDM by enhancing accessibility services, continuity, integration and quality through interorganizational collaboration.	Local health network (LHN) of PHC organisations from 2004: merger of community health centres/services, long-term care facilities, and acute/specialist hospitals; contractual agreements extended to all family doctors, i.e. private practices outside family medicine groups (FMGs); Nurses appointed for FMG; centralized information system including waiting lists; walk-in clinics; extended hours, on-call; centralized specialist services; CDM programme with interdisciplinary care, e.g. Diabetes Reference Centre – lifestyle change and education and treatment) – renamed CD Action Centre for expansion to CVD.	A review of published papers specific to Canada	Increase in PTs attached to GP from newly centralized waiting lists (n = 600,000). Increased PT use of Diabetes Reference Centre (n = 800 p.a). LHNs have facilitated shared local leadership, planning of care and access to specialist and PT education services. Centralized IT system not yet formally evaluated. No notable improvements in PT care experiences were noted.
Canada (Alberta, Ontario), Very high HDI Health Council of Canada, 2009^25^ Commissioned report on service case studies	To provide effective, accessible and sustainable healthcare for CDM through team-based PHC.	Collaborative PHC teams: targeted funding for CDM: self-management programmes and expansion; interdisciplinary clinics; specialist clinics; community-based nurses for CDM and specialist nurses); integrated information systems.	Baseline contextual survey data for each province followed by interviews nd additional documentation	Improved information sharing, e.g. PT data, clinical guidelines. Improved glycaemic × 17% and triglyceride × 13% control. Positive COPD PT behaviours e.g.smoking cessation × 15%; increased exercise × 30%. Decreased hospital admissions for COPD (19%) and for all PTs with CD × 41%. Decreased ED visits × 34%. Drivers of success: strong leadership; information systems; PT-centred partnership with professionals; interdisciplinary teams; continuous quality improvement approach; education and training of staff on CDM; Challenges/Barriers: shift from medical nd hospital model of care to PT self-management; need for cross-referral mechanisms between CDM programmes; understaffing and increased workloads; physician champions; need to grow nurse-led clinics; lack of specialty leadership; funding investment in IT systems and CDM programmes.
Slovenia, Very high HDI, World Health Organization, 2020^26^ Commissioned report on national health system case study.	Development of people-centred integrated PHC to reduce inequalities nd increase access to healthcare with a population health focus for people with chronic diseases.	People-centred integrated PHC: network of community health centres (CHCs); health promotion centres (HPCs) created within CHCs since 2002; co-location of CHCs with specialist services; expansion of family medicine team (FMTs) and multidisciplinary teams including community nurses and nurse practitioners (NPs) for CDM; CDM programmes; integrated PHC with public health, emergency and diagnostics; revised contractual agreement on capitation limits since 2018; financial incentives to family medicine practices to reach targets. Noted that CHCs have ensured universal health coverage as part of the wider health system.	Articles and reports on the Slovenian health system; health indicators from international databases; interviews with various stakeholders, e.g. public health & health system experts and health providers. CHCs site visits.	PHC performing well. High rates of screening programmes have led to a decline in CD premature mortality rates. Success factors: universal health care; expansion of FMTs with NPs led to improved diabetes monitoring; integration of PHC and public health. Barriers/Challenges: PHC provider high workloads and inadequate remumeration; inadequate PHC organizational and governance structures; inadequate quality improvement mechanisms, e.g. clinical guidelines; inadequate government funding to healthcare.

CD: chronic disease; CDM: chronic disease management; ER: emergency room; GP: general practitioner; LHNs: local health networks; PHC: Primary Healthcare; PT/s: patient/s

### Quality assessment

We assessed the quality of studies^19,21,22^ and a narrative review^20^ using relevant appraisal tools^27,28^ which are reported in Tables 2,3 and 4. Quality assessment was not applicable for the two case studies^23,25^ and the government report^24^ since to date there are no published quality assessment criteria for PHC service case examples that are not reported as studies, and likewise regarding commissioned reports. Although the commissioned reports presented a case study on a national health system,^26^ existing quality appraisal tools were not applicable because of a focus on clinical case–control studies^27,29^ rather than system level cases.

**Table 2.** Quality assessment summary of before–after study.

Criteria	Pineault et al.^22^
Clear study question or objective	Y
Prespecified selection criteria	Y
Sample representation	Y
Enrolment of all eligible participants that met inclusion criteria	Y
Confidence in sample size	CD
Service/intervention described nd consistently delivered	CD
Outcome measures – reliable, valid and used consistently	Y
Blinding of outcome assessors (detection bias)	NR
Attrition reported and accounted for	NA
Statistical reporting of changes in outcome measures including *p*-values	Y
Multiple measurements of time points	N
Use of individual-level data to determine effects of group-level data (i a group, i.e. whole system intervention)	Y

CD: Cannot determine; N: No; NA: Not applicable; NR: Not reported; Y: Yes.

**Table 3.** Quality assessment for observational cohort and cross-sectional studies.

Criteria	Atun et al.^19^ (2016)	Reeve et al.^21^ (2015)
Clear research question or objective	Y	Y
Population clearly specified and defined	Y	Y
Participation rate of eligible persons at least 50%	N/A	N/R
All the subjects selected or recruited from the same or similar populations	Y	Y
Providing size justification, power description or variance and effect estimates	NA	NA
Exposure(s) of interest measured prior to the outcome(s) being measured	Y	N
Timeframe sufficient so that one could reasonably expect to see an association between exposure and outcome	Y	N
Examine different levels of the exposure as related to the outcome	NA	Y
Measure the exposure clearly defined, valid, reliable, and implemented consistently across all study participants	N	N
Assess the exposure(s) more than once over time	N	N
Measure the outcome clearly defined, valid, reliable, and implemented consistently across all study participants	Y	Y
The outcome assessors blinded to the exposure status of participants	NR	NR
Loss to follow-up after baseline 20% or less	NR	NR
Key potential confounding variables measured and adjusted statistically for their impact on the relationship between exposure(s) and outcome(s)	Y	NR

CD: Cannot determine; N: No; NA: Not applicable; NR: Not reported; Y: Yes.

**Table 4.** Quality assessment for narrative review (Nicholson et al. 2012^20^).

Items	Score
(1) Justification of the article's importance for the readership	2
(2) Statement of concrete aims or formulation of questions	2
(3) Description of the literature search	0
(4) Referencing	2
(5) Scientific reasoning	0
(6) Appropriate presentation of data	0
sum score	6

The importance is not justified = 0. The importance is alluded to, but not explicitly justified = 1. The importance is explicitly justified = 2.

The data for quality assessment were extracted by MA and cross-checked by CB. Quality assessment was discussed between all four reviewers to reach a consensus where differences arose.

## Results

In total, eight articles are included in this analysis. In this section, the results are presented on PHC reforms under the following headings: characteristics of the studies/reports reviewed; the vision of PHC reform; features of PHC reform; and outcomes/impact of PHC reform.

### Characteristics of articles reviewed

As presented in Table 1, of the eight articles reviewed, evaluation of PHC health system reforms were reported from four countries with high^19^ or very high ^21–26^ HDI. The majority of articles reported on either Australia^20,21,24^ or Canada.^22,23,25^ The accounts of sampling varied across articles and for some was not explicit, therefore, making it difficult to determine the total population size of patients with chronic diseases represented in our review. Large populations sizes of patients were reported in three articles^19,21,22^ which ranged from almost 3000 residents from multiple sources^21^ to over half a million patients sourced from health records.^19^ Sampling also involved PHC activities, but the sample size was reported for Estonia only which involved over 35 million PHC records.^19^ Of the eight articles reviewed, evaluations of PHC reform were conducted in three studies designed as observational cohort,^19^ cross-sectional^21^ and before–after study.^22^ The remaining articles included a narrative review of published empirical data,^20^ commission reviews^24^ including service case studies and examples^20,25^ and a health system study.^26^ Data sources for PHC reform evaluation varied across articles including PHC/service records and reports^19,26^ national statistical/health databases^1921^ or patient population records or surveys,^19–22,27^ interviews/surveys with various stakeholders (e.g. leaders, PHC practices),^22,25,26^ financial audits/reports,^20,24^ and published data in the literature.^20,23,26^ All eight articles addressed PHC within the context of multiple physical chronic conditions mostly referring to respiratory conditions,^19,22,24–26^ diabetes,^19–26^ cardiovascular^19–26^ and musculosletal^22,25,26^ conditions as a particular burden or to illustrate outcomes of PHC reform.

### Quality appraisal

The before–after study^22^ met most assessment criteria (Table 2). Although the sample size was large for both data collection time periods, power calculations were not reported, and so confidence in sample size could not be determined. Likewise, power calculations were not reported for the observational cohort and cross-sectional studies,^19,21^ results varied and details on some other criteria were not reported or could not be determined (Table 3). The quality assessment on the narrative review^20^ met three of the six criteria regarding the justification of the article's importance for readership, explicit aims and supporting references for arguments made. Quality criteria not met in this review related to a description of search strategy, scientific reasoning concerning the sources (e.g. study designs) and quality of evidence and the presentation of concrete outcomes data (Table 4). Caution is needed, however, regarding the last two criteria since the SANRA scale^28^ focuses on studies reporting on quantifiable clinical outcomes rather than policy literature which was included in Nicholson et al.'s review.^20^ Further field testing for validity has been recommended.^28^

### PHC reform vision

The vision of PHC reform in relation to CDM related to improving population health through increased PHC accessibility^20–26^ and greater equity of services^20,21,26^ including affordable and financially sustainable healthcare.^19,24,25^ There was an emphasis on developing an integrated PHC system^19–26^ with reference to shifting CDM from specialist hospital-based services to PC and community health services. Healthcare reforms towards strengthening PHC were also envisioned as facilitating continuity of care for patients with chronic diseases,^22–24^ as well as improving quality of care.^20,23^

### Features of PHC reform

Although countries varied in how they named the main focus of PHC reform (e.g. family medical-centred PHC,^19^ local health networks (LHNs),^21^ family medicine groups (FMGs)),^22^ these variations reflect differences in terminology across countries rather than substantive differences between the types of reforms being implemented. Our analysis revealed that features of PHC reform across countries were more similar than different. The main features of PHC reform seen across articles reviewed related to the funding of PHC, PHC organization and infrastructure and PHC services for CDM.

#### Funding of PHC

All articles reviewed addressed funding implications of PHC reform.^19–26^ Government funding reallocation from hospital services to PHC and additional funding allocation and incentives to strengthen PHC services within the overall national health system were reported.^19–26^ For example, new provider payment mechanisms were introduced involving capitation and performance-related payments to motivate family doctors towards CDM in Estonia.^19^ Funding through commissioning and contractual agreements with PC providers was reported for Australia,^20,21^ Canada^22,23^ and Slovenia.^26^ For some countries, there was an explicit reference to targeted funding to support new initiatives such as implementation of Information Technology (IT) systems supporting data flow between sectors and access to electronic health records,^20,25^ expansion of PHC teams^19,20,25,26^ and the development and delivery of CDM programmes.^19–21,23,25,26^

#### PHC organization and infrastructure

Features of reform were found to relate to structural changes in the overall organization of PHC systems and to the implementation of infrastructural elements to support and strengthen PHC. There was evidence of health system reform evolving into new PHC organizational structures over time. For example, in Canada, Breton et al.^23^ reported on the merger of community health centres, acute/specialist hospitals and long-term care facilities towards an integrated health system that provided population-based accessible services. This merger incorporated existing FMGs and LHNs.^23^ The organization of PHC into networks or collaborative structures focusing on integrated and accessible services was also evident for Australia,^20,21,24^ and Slovenia.^24^ In Slovenia, PHC was organized around a network of community health centres that were co-located with specialist services.^26^ In Australia, the model of Divisions of GPs expanded to larger Medicare Locals^20,24^ from 2011 as part of the national PHC strategy.^20^ Since the establishment of larger Medical Locals in Australia, a new model of PHC has been introduced named primary health networks (PHNs) aimed at integrating PC and public health.^30^ While articles specific to evaluating PHNs within the context of CDM were not identified in our search strategy, PHNs will be raised later in the discussion section of this review. In addition, a partnership model for comprehensive PHC services was reported by Reeves et al.^21^ for the aboriginal community with reference to different sectors coming together, namely PHC, community and hospital services. This partnership included a joint governance structure for the allocation of funding across the sectors.^21^

The implementation of IT systems to support service integration across sectors was reported for Estonia,^19^ Australia^20^ and Canada.^23^ A total of 265 IT systems were described as centralizing patient waiting lists^23^ and facilitating the flow of patient data and information exchange between sectors and healthcare professionals.^19^ There was no reference to implementing information systems for Slovenia,^26^ a problem raised later when discussing the findings of the case study evaluating this country's PHC reform. Other infrastructural changes reported as part of PHC reform were expansion of multidisciplinary workforce in PHC,^19–21,24–26^ particularly nursing^19,20,23,25,26^ and service schemes such as extended or after hours,^20,22,23^ on-call and walk-in clinics.^23^ These service schemes were introduced to improve continuity of care and increase accessibility to services. Accessibility and continuity of care was also a basis for integrating specialist services into CDM clinical care in PHC settings.^21–23,25^

#### PHC services for CDM

The findings presented above in relation to PHC organization and infrastructure provide insights into how health system reforms were intended to improve the management of chronic diseases at the point of service delivery by shifting CDM from hospital-based specialist services to patients accessing clinical care and support in the community. The descriptions of PHC reform for CDM across all countries indicated that GPs/family physicians primarily led out on and were at the core of PHC services. For example, family medicine was described as being at ‘the heart of primary health care’ in Estonia.^19^ Likewise, the role of GP/family physician in Australia was described as critical to an effective PHC system that oriented healthcare away from hospital services,^24^ and the expansion of PHC centres was GP led.^20^ In Canada^23^ and Slovenia,^26^ PHC reform involved a shift towards formal enrolment of patients with family physicians in FMGs,^23^ and positioning family medicine physicians as the first point of contact for services.^26^

Although GPs/family physicians were noted to be at the centre of services in PHC reform, it was evident that other health professionals were seen as integral to CDM. An interdisciplinary approach involving multiple professionals for CDM was described as a feature of reform in all countries.^20,25,26^ apart from Estonia which referred to nurses only.^19^ Nurses were specifically referred to as having a key role in CDM working in family medicine/GPs as family^19^/practice nurses,^20,23^ or as community nurses delivering services in community health centres.^25,26^ Furthermore, the provision of specialist services for CDM was reported with reference to healthcare providers having access to diagnostic services and medical specialists in PHC settings.^21–23,25^

There was evidence of targeted programmes developed for CDM in PHC with reference to a government strategy to address the burden of disease.^19–21,25,26^ Our analysis revealed that these programmes were developed for individual diseases^20,21,23,25,26^ with diabetes most commonly targeted first for implementing CDM in PHC.^20,23,25^ For example, in Quebec, Canada, a Diabetes Reference Centre to support CDM subsequently evolved to a Chronic Disease Action Centre to include patients with cardiovascular disease.^22^ Some articles referred to these programmes being supported by quality improvement initiatives such as a quality bonus system to engage family doctors,^19^ quality improvement care collaborations^20^ and evidence-based clinical guidelines for managing individual diseases.^19,20,25^

### Evaluation findings

For this review, we categorized the evaluation findings regarding national PHC reforms for CDM into the following broad areas: funding and financial costs; PHC organization and infrastructure; PHC service utilization; CDM programme delivery; and patient outcomes. These areas were identified by categorizing the findings reported across articles.

#### PHC funding and financial costs

Evaluation findings in relation to funding or associated financial costs were reported in four articles.^21,25–27^ Apart from one article,^21^ there was evidence that funding allocation to support PHC reform was inadequate. For example, the actual budget allocated for PHC reform in Australia fell short of the planned budget.^24^ The development and implementation of CDM programmes was a challenge in Slovenia and Canada due to inadequate funding,^25,26^ Despite the vision for the implementation of a national information system in PHC in Slovenia, this was not realized due to inadequate allocation of government funding.^26^ Finally, at the level of general/family practice, funding incentives or remuneration for initiatives such as out-of-hours services were viewed unfavourably by GP/family physicians due to inconsistencies across regions, contracting complexities^24^ or inadequate funding for the services provided.^26^

#### PHC organization and infrastructure

Evaluation findings on PHC organization and infrastructure related to governance,^20,21,24–26^ information systems^20,21,23–26^ and workforce.^19–21^ Shared leadership at governance level was reported as a success factor in reforming PHC in Canada,^23,25^ whereas findings reported for Australia^20,24^ and Slovenia^26^ were not favourable. Weak governance structures to support PHC reform in Slovenia has impeded the decision-making authority of directors of community health centres.^26^ In Australia, the vision for an integrated health service between PHC and other sectors through Medicare Locals was not realized, and in the absence of shared governance, the services remained fragmented.^20,24^ The lack of or inadequate integrated information systems further contributed to fragmented services across some health sectors.^20,24,26^ Contrasting evidence was found in three articles relating to Canada^23,25^ and a remote region in Australia^21^ which reported increased clinical information sharing and improved accessibility to services when information systems were integrated as part of PHC reform.

Although workforce expansion as a key area of reform was reported in most articles,^19–21,24–26^ only two articles provided data on this expansion with reference to nursing. The narrative review article from Australia which was based on published data on PHC activities reported that 60% of GPs sampled in 2009–2010 employed practice nurses.^20^ In Estonia, it was reported that 27% of family medicine practices employed nurses.^19^ There was some evidence from other countries to suggest that the workforce to support PHC reform for CDM was under resourced.^25,26^

#### PHC service utilization

The impact of PHC reform on service utilization of people with chronic diseases was reported in five articles^19–23^ with mixed results evident. The expansion of practice nurses in Australia was reported to double patient consultations to GP practices.^20^ and likewise in Estonia, patient consultations to nurses in family medicine practices almost tripled.^19^ A trend towards increasing PHC service use and decreasing use of hospital services was also reported.^19,21,23,26^ The evaluation of FMGs with complementary network clinics in Canada was reported to yield unexpected results on service use.^22^ Pineault et al.^22^ found that following PHC reform, there was no significant change in emergency or hospital admissions, and that accessibility to PHC services decreased. An explanation for these findings offered by the researchers was that service use and care provision was investigated in relation to physicians only and not other healthcare professionals.^22^

#### CDM programme delivery

Outcomes reported on CDM programme delivery related to improvements in health assessment and patient monitoring.^21,26^ care planning^21,23^ and interdisciplinary collaborative approach to care.^20,21^ For example, an increase in health assessments seen in a partnership model between PHC and hospitals for an underserved community led to the majority of patients with diabetes being placed on care plans.^21^ Breton et al.^23^ also found that an interdisciplinary and intersectoral approach to delivering CDM facilitated care planning in patients with diabetes. Our analysis found little evidence of CDM programmes targeting chronic disease multimorbidity, a concern raised by Horvarth^24^ who was critical of the continued episodic approach to CDM in Australia following implementation of Medicare Locals in PHC. He argued that episodic care is inadequate in addressing complex multimorbidity associated with chronic diseases.

#### Patient outcomes

Evidence on patient outcomes related to clinical indicators of disease status (glycaemic control and triglycerides),^21,25^ lifestyle behavioural changes,^25^ mortality rates^26^ and patient perceptions or experiences of care.^22,23^ Positive outcomes were reported in terms of declining mortality rates in Slovenia attributed to a heightened emphasis on health promotion and CDM.^26^ Gylcaemic and triglyceride control as well lifestyle behavioural changes relating to smoking cessation and exercise were found to improve with team-based approaches to CDM.^25^ Patients’ perceptions of their care experiences indicated no improvements or changes following reforms following the expansion of family medical groups and network clinics in Australia^22^ (e.g. continuity of care, accessibility, unmet needs addressed) or for population-based PHC services for CDM in Canada.^23^

## Discussion

This systematic review was conducted to gain insight into the evidence from evaluations of PHC reforms for CDM implemented in countries with high or very high HDI. The findings indicate that between countries there were consistent motivating factors influencing PHC reform especially the need to shift away from specialist acute care for CDM. PHC reform at a population level is complex and fraught with challenges in relation to funding, governance, workforce, but a central factor is the engagement and involvement of GP/family doctors.

The vision to shift CDM away from specialist hospital services towards PHC as the first point of contact in the health system^19–26^ is consistent with the WHO's 2010 report urging the need to strengthen PHC systems to tackle the global burden of chronic diseases.^3^ The need to strengthen PHC within national health systems of countries continues to be highlighted by the WHO^5^ and other policy reports.^31,32^ Integrated PHC is a major focus of national health system reforms in countries such as the USA,^33,34^ the UK^34,35^ and other European countries^36^ intended to reduce service fragmentation and inefficiencies,^32,33,36^ a focus also evident in our review.^19–26^

Integrated healthcare systems are complex involving interorganizational (e.g. primary and acute care; health and social services, PC networks) and interdisciplinary team collaborations to facilitate patient care coordination and continuity at the point of service delivery.^37,38^ Structural changes towards interorganizational collaboration were evident in our review including Medicare Locals (Australia) as new meso-level organizations linking government and frontline healthcare providers,^20,24^ LHNs (Canada) as mergers of community health centres, acute/specialist services and long-term facilities^23^ and networks of community health centres co-located with specialist services in Slovenia.^26^ PHC reform through interorganizational collaborations requires consideration for governance, funding, information and human resources as key strategies towards health system integration.^34,38^ Of the countries reviewed, most evidence of positive influences of these strategies was reported from Canada through LHNs^23^ and collaborative PHC teams for CDM^26^ such as patient enrolment with GPs through centralized waiting lists,^23^ shared information/care planning^23,26^ and increased access to PHC for CDM including access to specialist care.^23,26^

Although some positive evaluations were found for Australia^20,21^ and Slovenia^26^ such as greater GP engagement in collaborative CDM programmes^21^ and increased use of PHC services for CDM,^20,26^ multiple barriers impeding interorganizational collaborations^38^ were reported. These were a lack of shared governance and leadership,^20,24,26^ inadequate funding and funding inefficiencies and inequities,^24,26^ lack of stakeholder engagement^24,26^ and inadequacy of information systems to support data sharing between organizations and healthcare professionals.^20,24,26^ In Australia, these barriers impeded Medicare Locals in achieving integration leaving services for CDM fragmented,^24^ a finding also reported about Medicare Locals concerning the broader context of health services.^29^ Medical Locals have since been replaced by regional PHNs involving integration between PC and public health sector through shared goals and infrastructures, and close alignment with hospital networks.^30^ In noting the multiple barriers herein, it must be acknowledged that the evidence is drawn from a small number of articles.

Integrating PC and public health is viewed as critical to shifting beyond curative and rehabilitative CDM to promotive and preventative services^30^ and is more aligned to the broader concept of PHC within which PC is a component.^12–14^ In our review, apart from Slovenia,^26^ explicit references to PHC reform involving the integration of PC and public health was lacking. A further gap concerning PHC reform for CDM relates to multimorbidity. The focus of CDM programmes on individual chronic conditions^20,21,23,25,26^ fails to take account of the increasing burden of multimorbidity on the health system and the challenges this presents to PHC providers working within a fragmented system with little integrated specialist support.^39,40^ A positive feature of PHC reform seen in our review was the expansion of multidisciplinary teams in PHC^20,21,23–26^ which can lead to integrated approaches to managing multimorbidity when these teams are located in the same PC practice as GPs.^39^

In multistakeholder health systems, governance is viewed as an important ‘functional block’ to ensuring shared goals, policymaking and financing because of these impacts on PHC delivery and outcomes.^39^ Apart from a regional partnership model from Australia involving a single governance structure and which reported increased funding allocation to PHC over time,^21^ there was little evidence of shared governance found in our review. In the absence of integrated governance, funding and financing mechanisms will not be aligned to meet health service needs to the detriment of PHC delivery in general and CDM,^39^ leaving healthcare providers dissatisfied with funding allocation, payment mechanisms and incentives which in turn runs the risk of PHC providers disengaging from health system reforms.^24,26^ The need for engagement with GPs/family physicians as a priority in reforming PHC was recommended by authors in our review.^19,20,23–26^ In this regard, lessons could be learned from the UK experiences of implementing Clinical Commissioning Groups involving GPs with decision-making authority concerning service provision, and flexible budgeting for service needs.^34^

## Strengths and limitations

A strength of this review is that a systematic approach was applied to searching, extracting, analysing and synthesizing the evidence. A challenge in undertaking the review was the heterogeneity of articles and how PHC reform was evaluated. Complex system-wide projects such as health service reforms are challenging to evaluate for researchers and this also applies to reviews of policy implementation.^41^ The rigour applied to our approach helped identify the variations and consistencies across PHC reforms and the pattern of outcomes relating to the impact of these reforms. The inclusion of grey literature is a strength by providing a more balanced account of the evidence inclusive of negative findings therefore minimizing publication bias.^16^ This review has some limitations. Only articles published in the English language were included, therefore, possibly missing evidence published in other languages. We included narrative reports as well as primary studies, therefore, yielding a heterogeneous selection of articles. Meta-analysis was not feasible and some articles were not eligible for quality assessment. A further limitation is that our focus was on physical chronic conditions to the exclusion of mental health conditions. The review is also limited by the exclusion of articles on single chronic conditions workforce articles that may have given additional insight into the PHC reform for chronic conditions. Given the vision for integrated PHC reform evident across countries, future reviews need to include mental health conditions and services.^42,43^

## Conclusions

Our review showed a consistent trend in the vision for healthcare system reforms from predominately hospital-centric to PHC services towards an integrated health system for the management of populations with chronic conditions. This shift towards an integrated PHC system involving interorganizational collaboration such as network organizations is becoming an ambition of many countries worldwide. However, healthcare systems are complex. Unlike a clinical intervention which can be evaluated through a randomized controlled trial in terms of its effects on outcomes, health system reform is by far more challenging to evaluate. The breadth and scope of reform, as well as a lack of knowledge on the most appropriate methodologies, contribute to the complexities of health system evaluation. Notwithstanding these challenges, our review offers insights into the progress being made across a select number of countries including successes and barriers. It is evident that integrated PHC reform for CDM involving interorganizational collaboration requires shared governance, shared decision-making around funding and payment mechanism, multidisciplinary team expansion, and an integrated information system. GPs as clinical leads of PC practice have a key role to play in PHC governance and their engagement at this level needs to be strengthened in countries where this is weak. This finding points to the importance of policymakers engaging with GP, as key stakeholders, with consideration to their views and experiences in shaping the future direction of PHC reform for managing chronic conditions.

## Supplemental Material

sj-doc-1-chi-10.1177_17423953211059143 - Supplemental material for Primary healthcare reform for chronic conditions in countries with high or very high human development index: A systematic review Click here for additional data file. Supplemental material, sj-doc-1-chi-10.1177_17423953211059143 for Primary healthcare reform for chronic conditions in countries with high or very high human development index: A systematic review by Mohammed Alyousef, Corina Naughton, Colin Bradley and Eileen Savage in Chronic Illness

sj-docx-2-chi-10.1177_17423953211059143 - Supplemental material for Primary healthcare reform for chronic conditions in countries with high or very high human development index: A systematic review Click here for additional data file. Supplemental material, sj-docx-2-chi-10.1177_17423953211059143 for Primary healthcare reform for chronic conditions in countries with high or very high human development index: A systematic review by Mohammed Alyousef, Corina Naughton, Colin Bradley and Eileen Savage in Chronic Illness

## References

[1] Hansen J Groenewegen PP Boerma WGW , et al. Living in a country with a strong primary care system is beneficial to people with chronic conditions. Health Aff 2015; 34: 1531–1537. 10.1377/hlthaff.2015.0582 26355055

[2] *World Health Organization* . Noncommunicable diseases, https://www.who.int/news-room/fact-sheets/detail/noncommunicable-diseases (2018, accessed 14 January 2019).

[3] *World Health Organization* . Global status report on noncommunicable diseases 2010, https://www.who.int/nmh/publications/ncd_report2010/en/ (2011, accessed 14 January 2019).

[4] Hone T Gurol-Urganci I Millett C , et al. Effect of primary health care reforms in Turkey on health service utilization and user satisfaction. Health Policy and Plan 2017; 32: 57–67. 10.1093/heapol/czw098 27515404

[5] *World Health Organization* . WHO European Centre for Primary Health Care: annual report of activities, WHO Regional Office for Europe, Copenhagen, http://www.euro.who.int/__data/assets/pdf_file/0004/373027/gdo-report-2018-eng.pdf?ua=1 (2017, accessed 1 March 2019).

[6] *Commonwealth of Australia* . National primary health care strategic framework, https://www.health.qld.gov.au/__data/assets/pdf_file/0027/434853/nphc_strategic_framework_final.pdf (2013, accessed 1 March 2019).

[7] *Government of Alberta* . Alberta's primary health strategy, https://open.alberta.ca/dataset/9781460108635 (2014, accessed 1 March 2019).

[8] *Health Council of Canada* . Primary health care: a background paper to accompany health care renewal in Canada: accelerating change, https://healthcouncilcanada.ca/files/2.48-Accelerating_Change_HCC_2005.pdf (2005, accessed 1 April 2019).

[9] Buenz EJ . Developed countries should be the focus for effectively reducing chronic disease. J Epidemiol Community Health 2006; 60: 562–563. 16790823 10.1136/jech.2005.043737 PMC2566226

[10] Khazaei Z Goodarzi E Borhaninejad V , et al. The association between incidence and mortality of brain cancer and human development index (HDI): an ecological study. BMC Public Health 2020; 20: 1–7. 33183267 10.1186/s12889-020-09838-4 PMC7664078

[11] *United Nations Development Programme* . Human Development Report 2016. Human Development for Everyone, http://hdrundp.org/sites/default/files/HDR2016_EN_Overview_Web_0.pdf (2016, accessed 5 November 2020).

[12] Muldoon LK Hogg WE Levitt M . Primary care (PC) and primary health care (PHC): what is the difference? Can J Public Health 2006; 97: 409–411. 17120883 10.1007/BF03405354 PMC6976192

[13] Félix-Bortolotti M . Part 1–unravelling primary health care conceptual predicaments through the lenses of complexity and political economy: a position paper for progressive transformation. J Eval Clin Pract 2009; 15: 861–867. 19811601 10.1111/j.1365-2753.2009.01274.x

[14] Awofeso N . What is the difference between ‘primary care’ and ‘primary healthcare’? Qual Prim Care 2004; 12: 93–94.

[15] Moher D Liberati A Tetzlaff J , et al. Preferred reporting items for systematic reviews and meta-analyses: the PRISMA statement. PLoS Med 2009; 6: e1000097. 19621072 10.1371/journal.pmed.1000097 PMC2707599

[16] Godin K Stapleton J Kirkpatrick SI , et al. Applying systematic review search methods to the grey literature: a case study examining guidelines for school-based breakfast programs in Canada. Syst Rev 2015; 4: 1–10. 26494010 10.1186/s13643-015-0125-0 PMC4619264

[17] Wei X Li H Yang N , et al. Changes in the perceived quality of primary care in shanghai and shenzhen, China: a difference-in-difference analysis. Bull World Health Organ 2015; 93: 407–416. 26240462 10.2471/BLT.14.139527 PMC4450701

[18] *United Nations Development Programme* . Human Development Report. The Rise of the South: Human Progress in a Diverse World, http://hdrundp.org/sites/default/files/reports/14/hdr2013_en_complete.pdf (2013, accessed 1 November 2019).

[19] Atun R Gurol-Urganci I Hone T , et al. Shifting chronic disease management from hospitals to primary care in Estonian health system: analysis of national panel data. J Glob Health 2016; 6: 020701. 27648258 10.7189/jogh.06.020701 PMC5017034

[20] Nicholson C Jackson CL Marley JE , et al. The Australian experiment: how primary health care organizations supported the evolution of a primary health care system. J Am Board Fam Med 2012; 25: S18–S26. 22403246 10.3122/jabfm.2012.02.110219

[21] Reeve C Humphreys J Wakerman J , et al. Strengthening primary health care: achieving health gains in a remote region of Australia. Med J Aust 2015; 202: 483–487. 25971572 10.5694/mja14.00894

[22] Pineault R Borgès Da Silva R Provost S , et al. Evolution of experience of care of patients with and without chronic diseases following a québec primary healthcare reform. Int J Chronic Dis 2016; 2016: 1–13. 10.1155/2016/2497637 PMC4838788 27144222

[23] Breton M Maillet L Haggerty J , et al. Mandated local health networks across the province of Québec: a better collaboration with primary care working in the communities. Lond J Prim Care 2014; 6: 71–78. 10.1080/17571472.2014.11493420 PMC4238724 25949720

[24] Horvath J . Review of Medicare locals: report to the Minister for Health and Minister for Sport. Department of Health, https://www1.health.gov.au/internet/main/publishing.nsf/Content/review-medicare-locals-final-report (2014, accessed 10 October 2018).

[25] *Health Council of Canada* . Getting it right: case studies of effective management of chronic disease using primary health care teams. Toronto: Health Council, http://publications.gc.ca/site/eng/430645/publication.html (2009, accessed 1 November 2018).

[26] *World Health Organization* . Integrated, person-centred primary health care produces results: case study from Slovenia, https://apps.who.int/iris/bitstream/handle/10665/336184/9789289055284-eng.pdf (2020, accessed 1 November 2020).

[27] *NIH National Heart, Lung, and Blood Institute* . Study quality assessment tools, https://www.nhlbi.nih.gov/health-topics/study-quality-assessment-tools (2018, accessed 15 February 2019).

[28] Baethge C Goldbeck-Wood S Mertens S . SANRA—A scale for the quality assessment of narrative review articles. Res Integr Peer Rev 2019; 4: 1–7. 30962953 10.1186/s41073-019-0064-8 PMC6434870

[29] Robinson S Varhol R Ramamurthy V , et al. The Australian primary healthcare experiment: a national survey of medicare locals. BMJ Open 2015; 5: e007191. 10.1136/bmjopen-2014-007191 PMC4386220 25818276

[30] Booth M Hill G Moore MJ , et al. The new Australian primary health networks: how will they integrate public health and primary care. Public Health Res Pract 2016; 26: 2611603. 26863166 10.17061/phrp2611603

[31] *European Commission* . Report of the expert panel on effective ways of investing in health (EXPH) on ttpology of health policy reforms and framework for evaluating reform efects, https://ec.europa.eu/health/sites/health/files/expert_panel/docs/013_healthpolicyreforms_reformeffects_en.pdf (2016, accessed 4 January 2021).

[32] *Department of Health and Social Care and the Ministry of Housing, Communities and Local Government* . Better Care Fund: Policy Framework, https://assets.publishing.service.gov.uk/government/uploads/system/uploads/attachment_data/file/821676/Better_Care_Fund_2019-20_Policy_Framework.pdf (2019, accessed 4 January 2021).

[33] Heeringa J Mutti A Furukawa MF , et al. Horizontal and vertical integration of health care providers: a framework for understanding various provider organizational structures. Int J Integr Care 2020; 20: 2.1-10 10.5334/ijic.4635 PMC6978994 31997980

[34] Oliver-Baxter J Bywood P Brown L . Integrated care: What policies support and influence integration in health care across New Zealand, England, Canada and the United States? Report 2. PHC RIS Policy Issue Review. https://www.researchgate.net/publication/257656562_ (2013, accessed 1 February 2021).

[35] Erens B Wistow G Mays N , et al. Can health and social care integration make long-term progress? Findings from key informant surveys of the integration pioneers in England. J Integr Care 2019; 28: 14–26.

[36] Borgermans L Devroey D . A policy guide on integrated care (PGIC): lessons learned from EU project integrate and beyond. Int J Integr Care 2017; 17:1-12. 10.5334/ijic.3295 PMC5854173 29588631

[37] Valentijn PP Schepman SM Opheij W , et al. Understanding integrated care: a comprehensive conceptual framework based on the integrative functions of primary care. Int J Integr Care 2013; 13: e010. 23687482 10.5334/ijic.886 PMC3653278

[38] Auschra C . Barriers to the integration of care in inter-organisational settings: a literature review. Int J Integr Care 2018; 18: 5. 1–14. 10.5334/ijic.3068 PMC5887071 29632455

[39] Rijken M Struckmann V van der Heide I , et al. How to improve care for people with multimorbidity in Europe? World Health Organization, https://www.ncbi.nlm.nih.gov/books/NBK464548/pdf/Bookshelf_NBK464548.pdf (2017, accessed 8 February 2021).

[40] Damarell RA Morgan DD Tieman JJ . General practitioner strategies for managing patients with multimorbidity: a systematic review and thematic synthesis of qualitative research. BMC Fam Pract 2020; 21: 1–23. 32611391 10.1186/s12875-020-01197-8 PMC7331183

[41] Lamont T Barber N de Pury J , et al. New approaches to evaluating complex health and care systems. Bmj. 2016; 352: i154. 26830458 10.1136/bmj.i154

[42] Moola S Munn Z Tufanaru C , et al. Chapter 7: Systematic reviews of etiology and risk. Joanna Briggs Institute Reviewer's Manual. The Joanna Briggs Institute, https://joannabriggs.org/sites/default/files/2019-05/JBI_Critical_Appraisal-Checklist_for_Case_Reports2017_0.pdf (2017, accessed 20 October 2019).

[43] Espinosa-González AB Delaney BC Marti J , et al. The impact of governance in primary health care delivery: a systems thinking approach with a European panel. Health Res Policy Sys 2019; 17: 65. 10.1186/s12961-019-0456-8 PMC6609383 31272472

