# Supplementary material for: Primary healthcare reform for chronic conditions in countries with high or very high human development index: A systematic review
Source: Chronic Illn. 2021 Nov 29;18(3):469–87. doi: 10.1177/17423953211059143 (PMC9397395; doi:10.1177/17423953211059143)
Supplement: sj-docx-2-chi-10.1177_17423953211059143 - Supplemental material for Primary healthcare reform for chronic conditions in countries with high or very high human development index: A systematic review [file sj-docx-2-chi-10.1177_17423953211059143.docx]

02/10/2021

Dear Editor

We would like to thank you for the opportunity to resubmit our third revised manuscript titled Primary Health Care Reform for Chronic Conditions in countries with high or very high human development index: A Systematic Review for your consideration for publication in the Chronic Illness. We would like to take this opportunity to express our thanks to the reviewers for the helpful comments for correction and modification. The manuscript has been revised and approved by all authors to address the reviewers' comments.

We confirm that this work is original and has not been published elsewhere, nor is it currently under consideration for publication elsewhere. It has never been published or under other consideration for publication elsewhere. We have no conflicts of interest to disclose.

We would like to note that the submission required ticking that we had received ethical approval, but this was not applicable to this paper. We are aware that the total number of words is over the words limit because of the complexity of this review and its heterogeneity. We are kindly asking you to consider the tables as supplementary data. We very much hope the revised manuscript is accepted for publication in Chronic Illness.
